# Cumulative expression of heterologous XlnR regulatory modules and AraR^A731V^ in *Penicillium oxalicum* enhances saccharification efficiency of corn stover and corn fiber

**DOI:** 10.1186/s13068-024-02464-x

**Published:** 2024-02-01

**Authors:** Chengqiang Xia, Xiaoyu Qi, Xin Song

**Affiliations:** 1 State Key Laboratory of Microbial Technology, Shandong University, Binhai Road 72, Qingdao, 266237 Shandong People’s Republic of China; 2 School of Municipal and Environmental Engineering, Shandong Jianzhu University, Jinan, 250100 Shandong China; 3 National Glycoengineering Research Center, Shandong University, Binhai Road 72, Qingdao, 266237 Shandong People’s Republic of China; 4 College of Animal Science, Shanxi Agriculture University, Minxiannan Road 1, Jinzhong, 030801 Shanxi China

**Keywords:** Filamentous fungi, XlnR regulatory modules, Cellulase, Hemicellulase, Transcription factor AraR

## Abstract

*Penicillium o*xa*licum* engineered strain DB2 and its mutant strains with multiple regulatory modules were constructed. Mutant strain RE-4-2 with two regulatory modules showed a significant increase in the reducing sugar released from corn stover and corn fiber as well as in the conversion of cellulose than DB2. RE-5-2 with three regulatory modules showed a further increase in reducing sugar released from corn stover and the conversion of cellulose on the basis of RE-4-2. RE-4-2-AraR^A731V^ constructed by overexpressing AraR^A731V^ in RE-4-2 showed an increase of 7.2 times and 1.2 times in arabinofuranosidase and xylosidase activities, respectively. Reducing sugar yield and cellulose conversion of corn stover and corn fiber by RE-4-2-AraR^A731V^ were further increased.

**Supplementary Information:**

The online version contains supplementary material available at 10.1186/s13068-024-02464-x.

## Background

Filamentous fungi such as *Trichoderma reesei* [1, 2], *Penicillium oxalicum* [3–5], *Neurospora crassa* [6, 7], are widely used in the production of industrial cellulases because of their ability to secrete complete and high level of lignocellulosic hydrolases. *T. reesei* RUT C30 is one of the most wildly used fungi for industrial cellulase production, its FPA (filter paper activity) reached 15 FPU/gdm [8], *P. oxalicum* RE-10 produced the maximum FPA of 12.69 U/mL in a 7.5-L stirred tank [9]. However, cellulases produced by present cellulase-producing fungi is still not high enough and thus production cost of cellulases is a major bottleneck in the development of lignocellulosic materials to high-value energy compound as ethanol, alternative food as artificial starch [10], etc. By genetically modifying transcription regulatory factors and cellulase genes, the regulatory network of lignocellulase expression in cellulase-producing filamentous fungi can be reconstructed. In filamentous fungi *T. reesei*, by overexpressing the gene Trvib-1 encoding a putative transcription factor in T. reesei Rut-C30, cellulase production in the culture of the recombinant *T. reesei Vib-1*, which were 200% higher than that produced by the parent strain [11], and in *P. oxalicum*, through the cumulative introduction of three regulatory modules containing regulator mutants and their corresponding target cellulase genes from *P. oxalicum*, *T. reesei*, and *N. crassa*, a 2.8-fold increase in cellulase production was achieved [12]. Therefore, the reconstruction of regulatory network of cellulases can improve the production of lignocellulosic degrading enzymes and thereby enhancing the hydrolysis efficiency of cellulose and ultimately lowering enzymatic hydrolysis cost of cellulose.

Our previous work demonstrated that overexpression of heterologous transcription regulatory factors Xyr1^A824V^ from *T. reesei* and XLR-1^A828V^ from *N. crassa* in *P. oxalicum* significantly increased cellulase and xylanase activities of *P. oxalicum* strain. When the regulatory modules containing Xyr1^A824V^ or XLR-1^A828V^ and their target genes were overexpressed in *P. oxalicum*, the activities of both exocellulase and endocellulase were enhanced [12]. In lignocellulosic biomass, cellulose and hemicellulose are intertwined to form a tense and complex structure, the removal of hemicellulose is benefit to the binding of cellulases to cellulose and enzymatic degradation of cellulose, thus, improving the degradation efficiency of hemicellulose is also effective to the efficient degradation of cellulose [13]. Wheat straw and corn stover are the most abundant and most widely studied raw materials, in their hemicellulose composition, L-arabinose content is second only to xylose, accounting for 2.5% and 2.9% of the two raw materials, respectively [14]. L-Arabinose mostly exists in the form of side chain substituent groups on hemicellulose, and the presence of these side chains prevents the binding of main-chain degrading enzymes to the main hemicellulose chain, thus affecting the degradation of hemicellulose. α-L-Arabinofuranosidase is able to hydrolyze these side chains on hemicellulose and has synergistic effects with xylanase and xylosidase [15].

In the present study, we aimed to investigate the saccharification efficiency of corn stover and corn fiber by lignocellulose-degrading enzymes of *P. oxalicum* strains in which multiple XlnR regulatory modules of different filamentous fungi were overexpressed. In addition, overexpression of AraR with a point mutation was expected to further enhance α-L-arabinofuranosidase activity and thus cellulase production.

## Methods

### Strains and culture conditions

The mutant M12 (a uracil auxotroph, *pyrG*^Q226*^) was derived from *P. oxalicum* wild-type strain 114-2 [16]. The strain 114-2 was deposited at the China General Microbiological Culture Collection Center (CGMCC) under the number of CGMCC 5302. M12 and all mutants constructed from this strain by our lab are listed in Table 1. The liquid glucose medium contained 1 × Vogel’s salts (Vogel 1956) and 2% (w/v) glucose. Wheat bran extract agar contained 10% wheat bran extract and 2% agar powder. Cellulose medium contained 1 × Vogel’s salts and 2% microcrystalline cellulose (Sangon Biotech, Shanghai, China). Xylan medium contained 1 × Vogel’s salts and 1% xylan (Sigma, St. Louis, USA). The complex carbon medium was composed of wheat bran (4.66%), corn cob residue (2.00%), soybean cake powder (1.00%), microcrystalline cellulose (0.60%), KH_2_PO_4_ (0.30%), NaNO_3_ (0.28%), (NH_4_)_2_SO_4_ (0.20%), urea (0.10%), and MgSO_4_ (0.05%). The protoplast transformation upper medium contained 18.2% sorbitol, 2% glucose and 1 × Vogel’s salts and 0.8% agarose, the protoplast transformation underlying medium contained 18.2% sorbitol, 1 × Vogel’s and 0.8% agarose. Transformation solution S1 contained 21.86% D-sorbitol, 1.36% KH_2_PO_4_, pH 5.6; Transformation solution S2 contained 18.22% D-sorbitol, 0.74% CaCl_2_, 0.48% Tris 4.84, pH 7.5; Transformation solution T1 contained 2.5% PEG, 0.74% CaCl_2_. 0.48% Tris 4.84, pH 7.5, pH 7.5. All the strains used in this study were cultivated on wheat bran extract agar at 30 °C for 3–5 days to harvest conidia. Fresh conidial suspension was added into a 100-mL liquid glucose medium in 300-mL flasks, and cultivated for 20 h to collect mycelia. The mycelia were transferred to a 50-mL cellulose medium for RNA extraction, or transferred to a 50-mL complex carbon medium for enzyme activity assays. All liquid cultures were cultivated in 300-mL flasks at 30 °C and 200 rpm in constant light. Uracil (0.5 g/L) was added to the culture of all uracil auxotrophic strains.

**Table 1:** *P. oxalicum* strains used in this study

Strain name	Description	Parent strain	Strain source
M12	*pyrG*^Q226*^	114-2	Qin et al., 2013
RE-3-1	Δ*Pbgl2-Pbgl2*(p)::*β-rec*; *PDE_02864*(p)::*PxlnR*^*A871V*^*-Pcbh1*(p)::*Pcbh1-Peg1*(p)::*Peg1-pyrG*	DB2	Previous work of our lab
RE-3-2	Δ*Pbgl2-Pbgl2*(p)::*β-rec*; *PDE_02864*(p)::*PxlnR*^*A871V*^*-Pcbh1*(p)::*Pcbh1-Peg1*(p)::*Peg1*	RE-3-1	Previous work in our lab
RE-4-1	Δ*Pbgl2-Pbgl2*(p)::*β-rec*; *PDE_02864*(p)::*PxlnR*^*A871V*^*-Pcbh1*(p)::*Pcbh1-Peg1*(p)::*Peg1*; *Pbgl2*(p)::*Txyr1*^*A824V*^*-Pcbh1*(p)::*Tcbh1-Peg1*(p)::*Teg1-pyrG*	RE-3-2	Previous work in our lab
RE-4-2	Δ*Pbgl2-Pbgl2*(p)::*β-rec*;*PDE_02864*(p)::*PxlnR*^*A871V*^*-Pcbh1*(p)::*Pcbh1-Peg1*(p)::*Peg1*;*Pbgl2*(p)::*Txyr1*^*A824V*^*-Pcbh1*(p)::*Tcbh1-Peg1*(p)::*Teg1*	RE-4-1	Previous work in our lab
RE-5-1	Δ*Pbgl2-Pbgl2*(p)::*β-rec*;*PDE_02864*(p)::*PxlnR*^*A871V*^*-Pcbh1*(p)::*Pcbh1-Peg1*(p)::*Peg1*; *Pbgl*_*2*_(p)::*Txyr1*^*A824V*^*-Pcbh1*(p)::*Tcbh1-Peg1*(p)::*Teg1*; *gpdA*(p)::*Nxlr-1*^*A828V*^*-Pcbh1*(p)::*Ncbh1-Peg1*(p)::*Neg1-pyrG*	RE-4-2	Previous work in our lab
RE-5-2	Δ*Pbgl2-Pbgl2*(p)::*β-rec*; *PDE_02864*(p)::*PxlnR*^*A871V*^*-Pcbh1*(p)::*Pcbh1-Peg1*(p)::*Peg1*; *Pbgl2*(p)::*Txyr1*^*A824V*^*-Pcbh1*(p)::*Tcbh1-Peg1*(p)::*Teg1*; *gpdA*(p)::*Nxlr-1*^*A828V*^*-Pcbh1*(p)::*Ncbh1-Peg1*(p)::*Neg1*	RE-5-1	Previous work in our lab
RE-4-2-AraR^A731V^	Δ*Pbgl2-P bgl2* (p)::*β-rec*; *PDE_02864*(p)::*PxlnR*^*A871V*^*-Pcbh1*(p)::*Pcbh1-Peg1*(p)::*Peg1*; *Pbgl2*(p)::*Txyr1*^*A824V*^*-Pcbh1*(p)::*Tcbh1-Peg1*(p)::*Teg1*; *gpdA*(p)::*Nxlr-1*^*A828V*^*-Pcbh1*(p)::*Ncbh1-Peg1*(p)::*Neg1*; *gpdA*(p):: *araR*^*A731V*^*-hph*	RE-4-2	Previous work in our lab

### Construction of engineered strains

The engineered strains DB2, RE-3-1, RE-3-2, RE-4-1, RE-4-2, RE-5-1, and RE-5-2 were constructed by our lab in previous work [9]. The knock-out cassette *Pbgl2*(p)::*β-rec*-*pyrG*-*bgl2* which was constructed by a double-joint PCR method was transformed into M12 to generate strain DB2-*pyrG*. The expression cassettes of regulatory modules as indicated in Table 1 were constructed by using the ExoCET direct cloning method [17]. The strains RE-3-1, RE-4-1, and RE-5-1 with *pyrG* were constructed by our lab in previous work. DB2-*pyrG*, RE-3-1, RE-4-1, and RE-5-1 were induced by 2% cellulose at 30 ºC and 200 rpm for 3 days. Single colonies grew on a glucose agar plate containing 5-fluoroorotic acid and uracil were selected for purification and verification, and the *pyrG* marker-free strains DB2, RE-3-2, RE-4-2, and RE-5-2 were obtained.

Construction of engineered strain RE-4-2-*araR*^*A731V*^. Expression cassette of *gpdA*(p)::*araR*^*A731V*^-*hph* was first constructed. The coding region of *araR* were amplified from the genome of *P. oxalicum* strain 114-2, mutation site was designed in advance in the sequence of the primers. Promoter *gpdA*(p) and hygromycin resistance gene *hph* were amplified using plasmid pAN7-1 and pSilent-1 as the template, respectively. Finally, these fragments were linked together by fusion PCR and the target expression cassette *gpdA*(p)::*araR*^*A731V*^-*hph* was obtained.

### Preparation and transformation of protoplasts

Protoplasts were prepared by the following procedure. *P. oxalicum* strain RE-4-2 was inoculated onto wheat bran slant, cultivated for 3–4 days at 30℃ until the slant was covered with a layer of brownish green spores. The conidial suspension was obtained by washing the spores on the slant with normal saline. A layer of cellophane was spread on sterile wheat bran agar medium, then 100 μL of fresh conidial suspension was added onto the cellophane and incubated for 12 h at 30 ºC. An appropriate amount of lyase was added into the conversion solution S1 to prepare 3% mycelium lysis buffer. Then, 3 mL of mycelium lysis buffer was added to a sterile plate, the mycelia on cellophane were put into mycelium lysis buffer and was covered with another layer of cellophane, 3 mL of lysis buffer was added between two layers of cellophane. The mycelia were lysed for 2 h at 30 ℃. The mycelia were scraped from the cellophane into the lysis buffer, then the remaining mycelia on the cellophane were rinsed with the conversion solution S1. The lysis buffer containing lysed mycelia was filtered using a funnel with three layers of lens paper, the filtrate was transferred into a 50-mL centrifuge tube, followed by centrifugation for 10 min at 2500 rpm. The supernatant was discarded and 4 mL of pre-cooled transformation solution S2 was added to the centrifuge tube and centrifuged for 10 min at 2500 rpm, 4 ℃. The supernatant was discarded again, and 400 μL of pre-cooled transformation solution S2 was added again. After mixing, the round and translucent protoplasts were prepared. Expression cassette *gpdA*(p)::*araR*^*A731V*^-*hph* was transformed into the prepared protoplasts of RE-4-2 by the following procedure. The reaction system contained 200 μL of protoplasts, 10 μL of *gpdA*(p)::*araR*^*A731V*^-*hph* and 50 μL of transformation solution T1. The above solutions were added to the test tube in order and completely mixed, and then placed on ice for 20 min. Two milliliters of transformation solution T1 was added into the reaction system, the test tube was gently shaken to thoroughly mix the solution, and stood at room temperature for 5 min. Four milliliters of transformation solution S2 was added to the reaction solution and mixed thoroughly to terminate the reaction. The reaction system was mixed with 30 mL of transformation upper layer medium containing 4‰ hygromycin B resistance at 40 ºC and added onto the transformation underlying medium. After the upper layer medium solidified, the plate was sealed and incubated at 30 ºC for 3–4 days. The transformants was purified and validated, the recombinant strain RE-4-2-*araR*^*A731V−*^*hph* was obtained, then RE-4-2-*araR*^*A731V*^ was obtained by cellulose induction.

### Selective marker removal induced by cellulose

The procedure of selective marker removal was as follows: firstly, 30 μL of conidial suspension (10^7^/mL) was inoculated into 5 mL liquid medium with 2% cellulose and induced at 30 ℃ and 200 rpm for 3 days. Mycelia were spread on a cellulose agar medium containing 5-fluoroorotic acid and uracil, and incubated at 30 ℃ for 3 days. Single colonies were picked and purified on a glucose agar plate containing 5-fluoroorotic acid and uracil. Single colonies were selected for purification and verification, and the strains with the removal of *pyrG* were obtained.

### RT-qPCR

RT-qPCR was carried out as the following procedure. The genomic DNA was first removed according to the instructions of PrimeScriptTM RT. Reaction system (10 μL) which contained gDNA Eraser 1 μL, 5 ×*g* DNA Eraser buffer 2 μL, RNase-free ddH_2_O 6 μL, total RNA 1 μL was incubated for 2 min in water bath at 42 ℃ and the reaction was ended on ice, the reaction solution with genomic DNA removal was obtained. Then RNA was transcripted to cDNA according to the following procedure. Reaction system (10 μL) which contained PrimeScript RT Enzyme MixI 1 μL, RT Primer Mix 1 μL, 5 × PrimeScript buffer 4 μL, RNase-free ddH_2_O 4 μL, reaction solution with genomic DNA removal 10 μL was incubated for 30 min at 37 ºC and the reaction was ended by placing the reaction system at 85 ºC for 5 s, and the sample was placed on ice or stored at − 20 ºC for use. After cDNA was synthesized, it was used as a template for fluorescence quantitative PCR. The reaction system for fluorescence quantitative PCR (10 μL) contained the follows: 2 × SYBR Premix Ex TaqII 10 μL, forward primer (10 μM) 0.8 μL, reverse primer (10 μM) 0.8 μL, cDNA 1.0 μL, ddH_2_O 7.4 μL. The above reaction system for RT-qPCR was performed on Roche Real-Time PCR instrument according to the following procedure: 1 cycle at 95 ºC for 2 min; 40 cycles at 96 ºC for 10 s; 61 ºC for 30 s; 80 ºC, plate read; 61 ºC for 5 min; Tm at 65–90 ºC, read the value every 0.5 ºC. The primers used in RT-qPCR are listed in Additional file 1: Table S1.

### SDS-PAGE analysis

The mixture of culture supernatants and 5 × loading buffers were boiled for 10 min and loaded onto a 12.5% polyacrylamide gel. Coomassie brilliant blue R250 (Sangon, Shanghai, China) was used for staining. Then, the protein gel was washed by a destaining solvent (methanol, acetic acid, and water, 1:1:8, v/v/v) until the background turned clear.

### Phenotypic observation of strains on different plates

#### Phenotype on glucose agar plate

Glucose agar medium with 2% glucose as the carbon source was prepared, then was cooled and placed at room temperature overnight to make the surface moisture volatilize and facilitate the subsequent plate experiment. The concentration of fresh spore suspension of *P. oxalicum* was adjusted to 10^7^ CFU/ml. 1.5 μL of spore suspension (10^7^ CFU/ml) were pipetted and slowly added to the phenotypic plate. After the spore suspension was completely absorbed by the medium, the plate was sealed and incubated at 30 ºC for 3–4 days, then phenotypes of strains were observed.

#### Phenotype on cellulose agar plate

The underlying agar medium without carbon source was first prepared, then the upper layer medium with 1% ball-milled cellulose (containing 1‰ Triton-100) and 1.5% agar was melted and then added onto the solidified underlying medium and placed at room temperature overnight. The spore suspensions (10^7^ CFU/ml) were inoculated onto cellulose plates and cultured for 4–6 days, then phenotypic observation was performed.

#### Phenotype on starch agar plate

Starch agar medium with 2% starch as the carbon source was prepared. Before the phenotypes of strains on starch agar plate were observed, iodine solution (6% KI and 0.6% I_2_) should be added onto the starch plate for 5 min, then the sizes of the starch hydrolytic halos could be observed.

### Enzyme activity assay

#### Amylase activity assay

1.5 mL of 1% starch solution and 0.5 L of diluted enzyme solution was added into 1.5-mL test tubes, and was placed in a water bath at 40 ℃ for 10 min, then 3 mL of DNS was added to terminate the reaction. The reaction solution was then boiled for 10 min and then cooled to room temperature, 20 mL of distilled water was added to the test tubes. After thorough mixing, 200 μL reaction solution was taken for determination of OD value under 540 nm.

#### Arabian furanosidase (pNPAase) activity assay

50 μL of 1 mg/mL pNPA solution was added into 1.5-mL centrifuge tubes, followed by the addition of 100 μL diluted enzyme solution, and then incubated in 50 ºC water bath for 30 min, 150 μL of 10% Na_2_CO_3_ was added to terminate the reaction. 200 μL reaction solution was applied to read OD value under 420 nm.

#### β-Glucosidase (pNGAase) activity assay

50 μL of 1 mg/mL pNPG solution was added into 1.5-mL centrifuge tubes, followed by the addition of 100 μL diluted enzyme solution, and then incubated in 50 ºC water bath for 30 min, 150 μL of 10% Na_2_CO_3_ was added to terminate the reaction. After thorough mixing, 200 μL reaction solution was applied to read OD value under 420 nm.

#### Filter paper enzyme (FPase) activity assay

Whatman filter paper was used for filter paper enzyme activity measurement. Filter paper was made into circular pieces with a diameter of 0.5 cm with a hole punch. Circular pieces of filter paper with a total mass of 50 mg was added to test tubes, then 1.5 mL acetic acid–sodium acetate buffer and 0.5 mL diluted enzyme solution were added. The reaction system was placed into a 50 ºC water bath for 1 h, then 3 mL of DNS was added to terminate the reaction. After the reaction solution was boiled for 10 min and cooled to room temperature, 20 mL of distilled water was added. After thorough mixing, 200 μL reaction solution was taken for OD value measurement under 540 nm.

#### Endocellulase (CMCase) activity assay

1.5 mL of 1% sodium carboxymethyl cellulose solution was added into test tubes, then 0.5 mL diluted enzyme solution was added and incubated in water bath at 50 ºC for 30 min, 3 mL of DNS was added to terminate the reaction. After the reaction solution was boiled for 10 min and cooled to room temperature, 20 mL of distilled water was added. After thorough mixing, 200 μL reaction solution was taken to measure OD value at 540 nm.

#### Xylanase activity assay

1% Betula xylan was used as the substrate for enzyme activity assay. The enzyme activity determination process was consistent with that of endocellulase activity.

#### Exocellulase (pNPCase) activity assay

50 μL of 1 mg/mL *p*NPC solution was added into 1.5-mL centrifuge tubes, then 100 μL diluted enzyme solution was added, and then incubated in a water bath at 50 ºC for 30 min. 150 μL of 10% Na_2_CO_3_ was added to terminate the reaction. After thorough mixing, 200 μL reaction solution was applied to read OD value under 420 nm.

#### Xylosidase (pNPXase) activity assay

50 μL of 1 mg/mL *p*NPX solution and 100 μL diluted enzyme solution were added into 1.5-mL centrifuge tubes, then incubated in a water bath at 50 ºC for 30 min, 150 μL of 10% Na_2_CO_3_ was added to terminate the reaction. After thorough mixing, 200 μL reaction solution was applied to read OD value at 420 nm.

### Active enzyme profiling

Profiling of active xylanase was conducted the same as profiling of active endocellulases. The substrate in the separation gel of SDS-PAGE was replaced with 0.5% xylan.

Profiling of active amylase was analyzed by adding 0.5% starch substrate into separation gel of SDS-PAGE. The steps of gel preparation, electrophoresis and renaturation are consistent with the analysis of the active enzyme profile of endocellulases, the difference is only that the enzymolysis of starch was conducted for 10 min at 50 ºC. Staining was carried out in iodine solution after enzymolysis.

No substrate was added to the separation gel for profiling of active β-glucosidase.

After the renaturation, the reaction was carried out at 50 ºC in 0.1% heparin (prepared with acetic acid–sodium acetate buffer of pH 4.8). The reaction could be terminated after the appearance of enzymolysis bands.

### Saccharification of corn stover and corn fiber pretreated with ammonium sulfite

The raw material of corn stover and corn fiber was subjected to ammonium sulfite pretreatment at 170 ℃ for 1 h in the mixture of 10% ammonium sulfate and 3% ammonium carbonate solution, with a solid–liquid ratio of 1:6. After the pretreatment, corn stover was rinsed by water until neutral, then dried, and ground into powder for use.

Saccharification effect of cellulase solution on corn stover and corn fiber pretreated with ammonium sulfite was evaluated. The strain was fermented in complex carbon source medium, and the fermentation broth after six days’ cultivation was used for saccharification experiment. Saccharification experiments were carried out in a 100-mL flask with 25 mL liquid loading. The proportion of glycosylated substrate was 2%, enzyme solution was added at a rate of 10 mg/g (protein/substrate), 0.2 M of pH 4.8 acetic acid–sodium acetate buffer and 0.1% sodium azide for bacteriostasis were added into the reaction flask, then the flask was sealed with tin foil:

$$\mathrm{Cellulose\, conversion\, }\left({\mathrm{\%}}\right)= \frac{\mathrm{The\, reducing\, sugars\, obtained\, by\, enzyme\, hydrolysis}}{\mathrm{Amount\, of\, pretreated\, corn\, stover\, and\, corn\, fiber\, used}\left({\text{mg}}\right)}\times 100{\mathrm{\%}}.$$

## Results and discussion

### Construction of high cellulase-producing strains

The strain DB2 with a rec/six screening marker reuse system was established by our lab previously, it has a relatively high production of cellulase and was used as the starting strain. First, the native regulatory module XlnR^A871V^ from *P. oxalicum* was constructed, consisting of three parts: transcriptional regulatory factor *PDE_02864*(p): P*xlnR*^A871V^, cellulase target genes P*cbh1*(p): P*cbh1* and P*eg1*(p): P*eg1*. Next, the native XlnR^A871V^ regulatory module of *P. oxalicum* was transformed into strain DB2 to obtain strain RE-3-1. After removing the screening marker gene *pyrG* from strain RE-3-1 through cellulose induction, the marker-free strain RE-3-2 was obtained. Further, the optimized regulatory module of *T. reesei* Xyr1^A824V^ (*Pbgl2*(p)::*Txyr1*^*A824V*^*-Pcbh1*(p)::*Tcbh1-Peg1*(p)::*Teg1-pyrG*) was transferred into RE-3-2 to obtain strain RE-4-1 with screening marker and strain RE-4-2 with screening marker removed. Finally, the XLR-1^A828V^ regulatory module of *N. crassa* (*gpdA*(p)::*Nxlr-1*^*A828V*^*-Pcbh1*(p)::*Ncbh1-Peg1*(p)::*Neg1-pyrG*) was transformed into strain RE-4-2 to obtain strain RE-5-1. After removing the screening marker in RE-5-1, strain RE-5-2 was obtained (Additional file 1: Fig. S1). After multiple genetic modifications, the following strains were obtained in sequence, RE-3-1, RE-3-2, RE-4-1, RE-4-2, RE-5-1, and RE-5-2. Among them, strains RE-3-2, RE-4-2, and RE-5-2 are *pyrG*-deficient strains, which are convenient for further genetic manipulations as starting strains. Therefore, various analyses were conducted on these three deficient strains.

### Phenotypes of high-yield strains on glucose, starch and cellulose agar plates

**Fig. 1:**
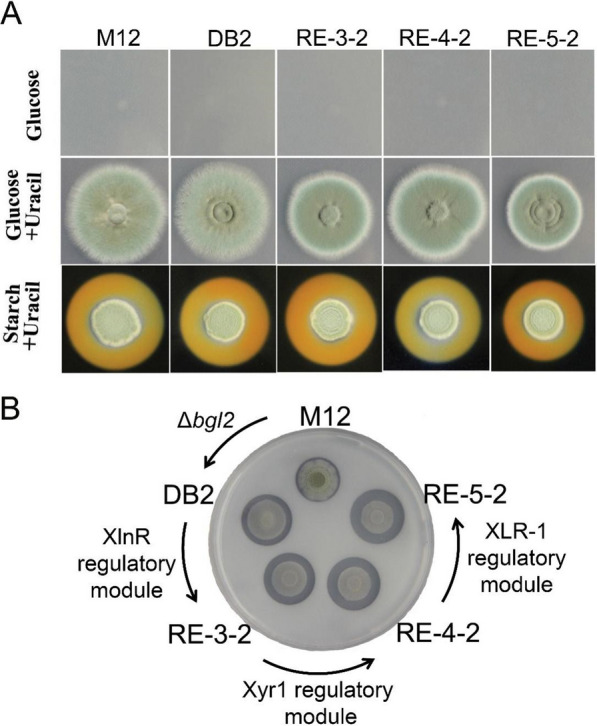
Phenotypic observation of M12 and its engineered strains on different plates. **A** Phenotypic observation of M12 and its engineered strains on glucose and starch plates. **B** Phenotypic observation of M12 and its engineered strains on 1% cellulose plates after 144 h of cultivation

**Fig. 2:**
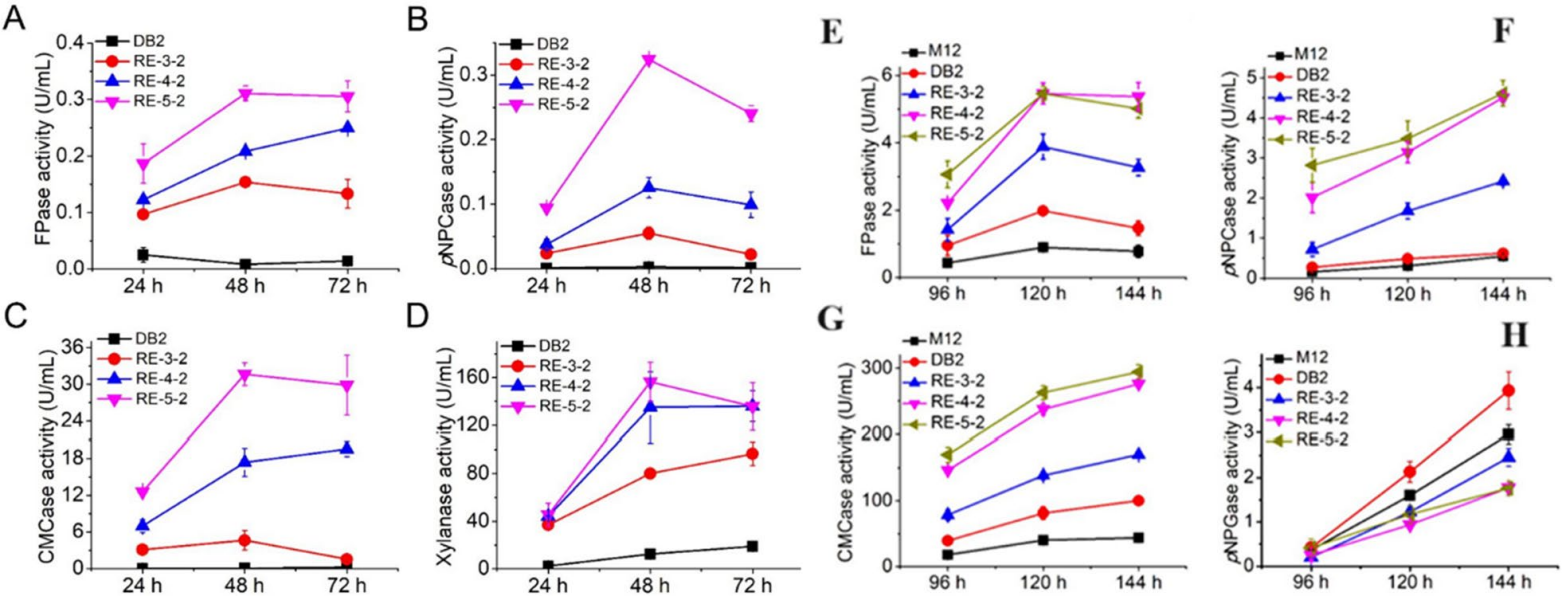
Activity analysis of cellulases and xylanases of recombinant strains in xylan medium and complex carbon source. **A**–**D** FPase, *p*NPCase, CMCase and xylanase production by xylan induction; **E**, **F** FPase, *p*NPCase, CMCase and xylanase production in complex carbon source [13]

The phenotypes of strains M12, DB2, RE-3-2, RE-4-2 and RE-5-2 on glucose plate are shown in Fig. 1. Strains M12, DB2, RE-3-2, RE-4-2 and RE-5-2 grew well in glucose media containing uracil, but cannot grow on glucose media without uracil, which indicated that *pyrG* gene were all deficient in the genome of these five strains. On starch plate, the sizes of starch hydrolysis halo of strains M12, DB2 and RE-3-2 were similar and larger than those of RE-4-2 and RE-5-2, which indicated that amylase activities of M12, DB2 and RE-3-2 were higher than those of RE-4-2 and RE-5-2. Next, the growth of the five strains on cellulose plates was observed. As seen in Fig. 2, the starting strain M12 had the smallest cellulose hydrolysis halo, strain DB2 (Δ*Pbgl2*::*Pbgl2*(p)-*β-rec*) that was obtained by deletion of gene *bgl2* and promoter P*bgl2* controlled overexpression of *β-rec* (coding *β-*recombinase) in M12 showed a lot increase in cellulose hydrolysis halo, indicating enhanced capability of degrading cellulose and inconsistent with the enzyme activity measurement results. Strains RE-3-2, RE-4-2 and RE-5-2 exhibited successively increased cellulose hydrolysis halos, which indicated that cellulose degradation ability of the three strains was gradually enhanced as the expression modules in the strain were cumulatively expressed.

### Lignocellulose-degrading enzyme production of strains by xylan induction

The presence of XlnR and its homologs in the three regulatory modules showed strong activation effect for the expression of xylanase by xylan induction. Therefore, the expression levels of lignocellulose-degrading enzymes were investigated by using xylan as the carbon source in fermentation medium.

As seen in Fig. 2, activities filter paper enzyme, endocellulase, and exocellulase of DB2 were at all at a low level, which indicated that xylan was not suitable carbon source for the production of lignocellulose-degrading enzymes of DB2. Stain RE-3-2 with one regulatory module from *P. oxalicum* showed significant increase in its cellulase activities. Compared with those of starting strain DB2, the activities of filter paper enzyme, exocellulase, endocellulase and xylanase of strain RE-3-2 were increased by 18.2, 18.4, 67.4 and 4.9 times, respectively. All lignocellulose-degrading enzyme activities of the recombinant strains RE-4-2 with double regulatory modules and RE-5-2 with three regulatory modules were also cumulatively enhanced as expected. This indicated that these regulatory modules were successfully expressed and played an activating effect in *P. oxalicum*. Stain RE-5-2 with three regulatory modules exhibited the highest lignocellulose-degrading enzyme activities under xylan induction condition. Activities of filter paper enzyme, exocellulase, endocellulase and xylanase were increased by 37.7, 113.6, 465.4, 11.5 times, respectively, when compared with those of strain DB2. Compared with the cellulase production of strain DB2, RE-3-2, RE-4-2, RE-5-2 in xylan medium, the cellulase production in complex carbon source medium were much higher. The cellulase activity of RE-3-2 with one regulatory module showed a significant increase, filter paper enzyme, exocellulase, and endocellulase activities increased by 1.0, 2.5, and 0.7 times compared to those of the starting strain DB2. The cellulase production ability of strain RE-4-2 with double regulatory modules was further enhanced, with filter paper enzyme activity, exocellulase activity, and endoglucanase activity increased by 0.4, 0.9, and 0.7 times compared to those of strain RE-3-2. Unlike other enzyme activities, β-glucosidase activity of RE-4-2 decreased by 44% compared with that of strain RE-3-2, which indicated that Xyr1^A824V^ has a significant inhibitory effect on the expression of β-glucosidase. Compared with strain RE-4-2, strain RE-5-2 with three regulatory modules showed a slight increase in exocellulase, and endocellulase activities, its β-glucosidase activity was almost the same as that of RE-4-2. Overexpression of transcription factors PxlnR^A871V^ or Nxlr-1^A828V^ could cause a significant decrease in the β-glucosidase activity [12]. These results indicated that complex carbon source was more favorable to cellulase production for these recombinant strains than that only using xylan as carbon source.

### Hemicellulase production by the recombinant strains in complex carbon source medium

**Fig. 3:**
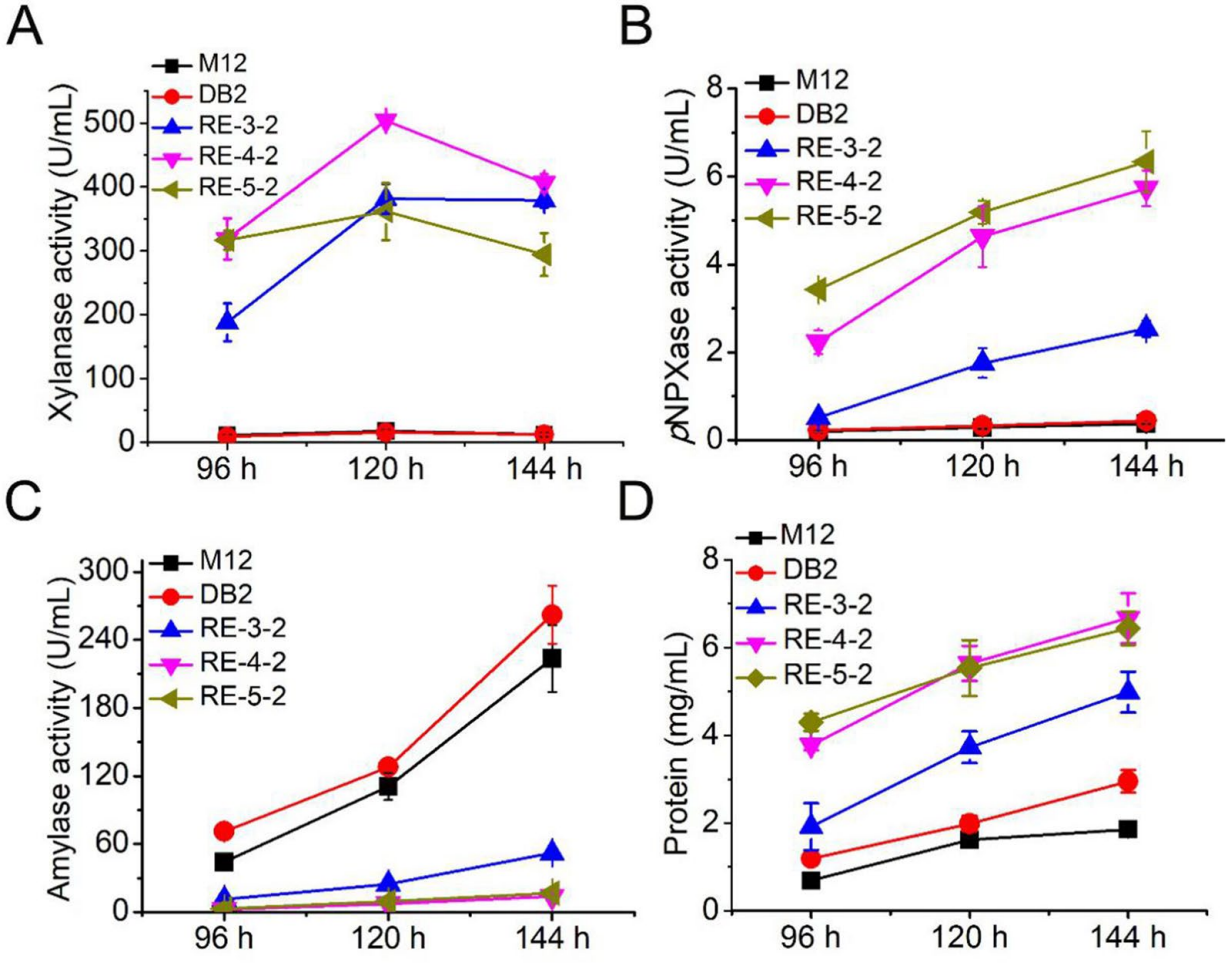
Hemicellulase, amylase activity analysis and protein content of five strains in complex carbon source medium. **A**–**C** Xylanase, *p*NPXase and amylase activities of five strains. **D** Extracellular protein content of five strains. Data represent mean ± SD from triplicate cultivations. Statistical significance of the differences between strains was calculated for transcriptional levels of endogenous genes. *p < 0.05, **p < 0.01, ***p < 0.001

Hemicellulase activities of the mutant strains were determined. As seen in Fig. 3, there was no significant difference in xylanase activity between strain DB2 and original strain M12, and both were at a very low level. Xylanase activity of strain RE-3-2 was about 20 times higher than that of strains M12 and DB2. Xylanase activity of strain RE-4-2 was further improved compared with that of strain RE-3-2, which was 28.0 times higher than that of strain M12. However, xylanase activity of strain RE-5-2 decreased slightly compared with that of strain RE-4-2. The reason for the decrease in xylanase activity of strain RE-5-2 was probably attributed to the significant lower transcription level of xylanase gene *Pxyn10A* of RE-5-2 than that of RE-4-2. There was no significant difference in xylosidase activity between strains M12 and DB2; both were at a low level. However, the xylosidase activities of the three strains RE-3-2, RE-4-2, and RE-5-2 increased sequentially, and were significantly increased compared to M12. Strain RE-5-2 exhibited the highest activity of xylosidase, was 17.2 times higher than that of M12. Based on the above results, it can be concluded that overexpression of the regulatory modules had a more significant effect on production of hemicellulases than cellulases, which was directly related to the regulatory characteristics of XlnR^A871V^/Xyr1^A824V^/XLR-1^A828V^ in the regulatory module. In addition, the amylase activity of strains RE-3-2, RE-4-2 and RE-5-2 decreased significantly.

### Analysis of extracellular proteins of different strains by SDS-PAGE and active zymogram

**Fig. 4:**
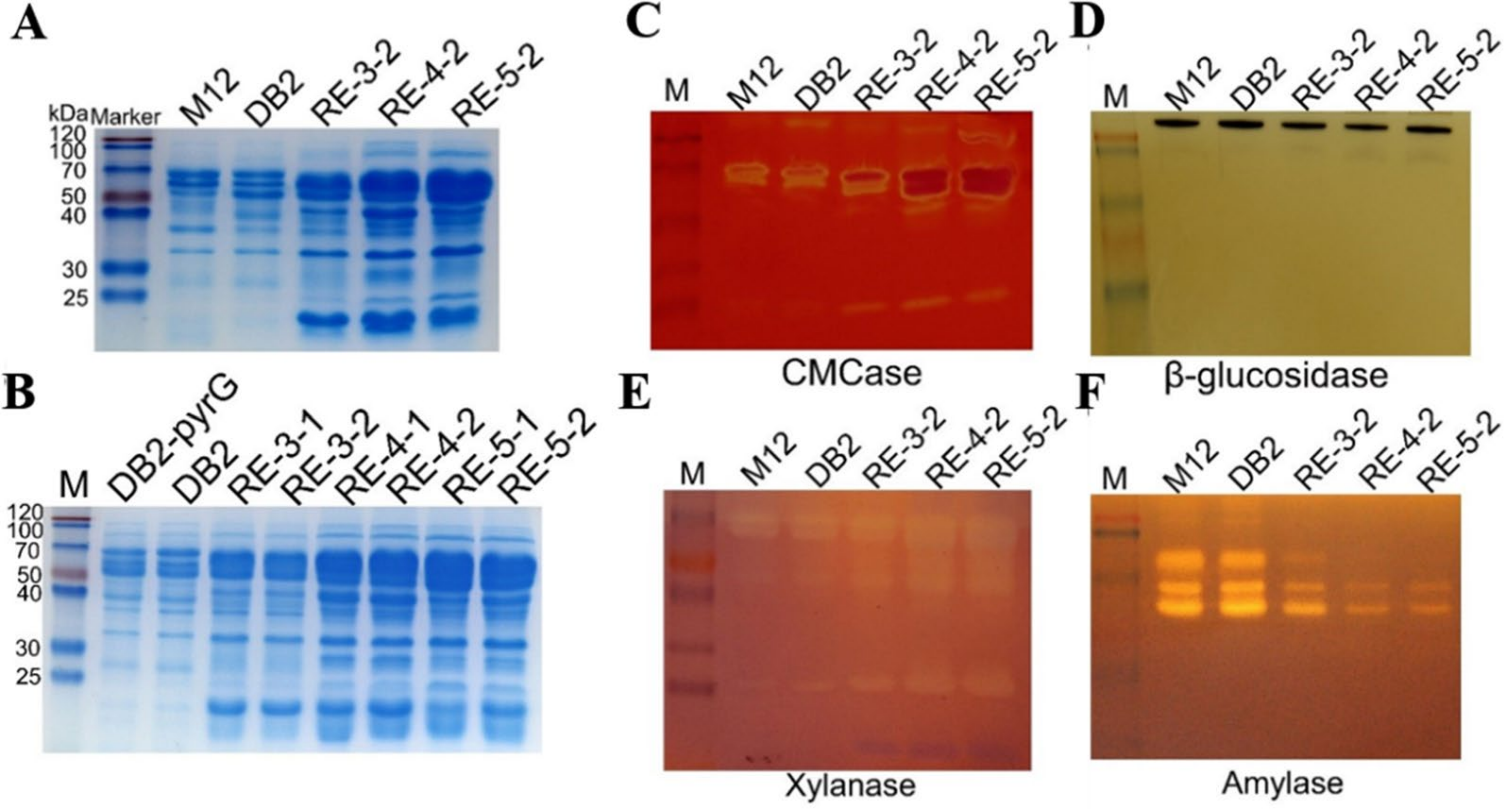
Analysis of extracellular proteins and CMCase, *p*NPGase, xylanase and amylase zymogram of different engineered strains after 120 h of cultivation. **A** SDS-PAGE of extracellular proteins of M12 and its engineered strains without *pyrG* marker. **B** SDS-PAGE of extracellular proteins of four engineered strains with *pyrG* marker and corresponding pyrG-free strains. Enzymatic analysis of fermentation broth of five strains. **C**–**F** Active zymogram analysis of CMCase, *p*NPGase, xylanase and amylase of M12 and its engineered strains

SDS-PAGE was used to analyze extracellular protein secretion and enzyme expression levels of these strains. As shown in Fig. 4, the strains with more modifications showed increased secretion of extracellular proteins, and increased in the order of M12, DB2, RE-3-2, RE-4-2 and RE-5-2. The concentration of extracellular proteins secreted by RE-5-2 was significantly increased. There is no difference in protein secretion of the strains with or without removal of the screening marker, indicating that the presence or absence of the screening marker gene *pyrG* did not affect extracellular protein secretion and lignocellulosic enzyme activity of these strains.

To more intuitively observe endocellulase, β-glucosidase, xylanase activities of high cellulase-producing strains constructed earlier, active zymogram of extracellular proteins of the recombinant strains was analyzed. As seen in Fig. 4, at least 5 endocellulases can be detected in the extracellular proteins of strains RE-4-2 and RE-5-2, and the concentrations was all higher than that of their starting strains M12, DB2 and RE-3-2; At least 5 xylanases can be observed in the extracellular proteins of strains RE-4-2 and RE-5-2, and their concentrations all increased compared with those of their starting strains M12, DB2 and RE-3-2; Different from endocellulases and xylanases, β-glucosidase and amylases of RE-4-2 and RE-5-2 showed a significant decrease. The results of enzyme activity profiles showed that the expression of endocellulase and xylanase increased gradually with the increase of genetic modifications, while the expression levels of amylase and β-glucosidase gradually decreased (Fig. 4). Overall, the above results demonstrated that the cumulative expression of XlnR regulatory modules from different sources in *P. oxalicum* can effectively increase the production of lignocellulosic degrading enzymes in the recombinant strains.

### Effects of point mutation of AraR on the lignocellulose degradation ability of high cellulase-producing strains

In *P. oxalicum*, AraR has a significant regulatory effect on the expression of arabinofuranosidase. Through homologous sequence alignment (Additional file 1: Fig. S2), our laboratory previously found that the amino acid sites of AraR^731^ and XlnR^871^ were relatively conservative and both were alanine (A), then alanine was mutated to valine (V). AraR^A731V^ with point mutation can increase α-L-arabinofuranosidase activity of the recombinant strain by 50 times, while the overexpression of AraR without point mutation has no significant effect on α-L-arabinofuranosidase activity [6]. Therefore, the regulatory factor AraR^A731V^ with point mutation were overexpressed on the basis of high-yield strain RE-4-2, hoping to improve the production of α-L-arabinofuranosidase, and thus can improve the degradation efficiency of hemicellulose and cellulose. The overexpression cassette of AraR^A731V^ were obtained through fusion PCR and was transferred into strain RE-4-2. After purification and validation (Additional file 1: Fig. S2), the overexpression strain RE-4-2-AraR^A731V^ with AraR^A731V^ was obtained.

### Enzyme activity analysis of recombinant strain RE-4-2-AraR^A731V^ overexpressing AraR with point mutation

**Fig. 5:**
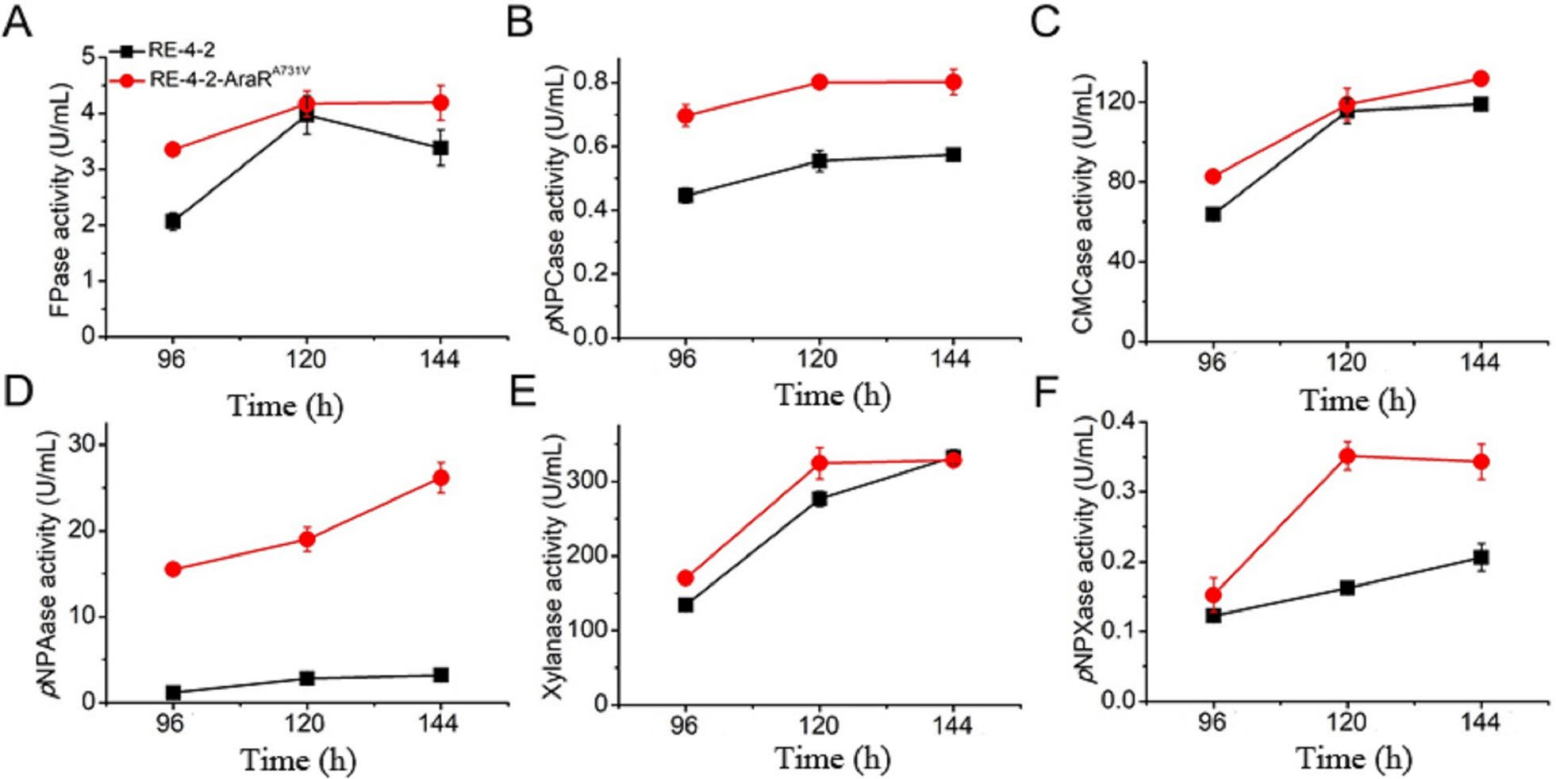
Lignocellulose degrading enzyme activity analysis of strains RE-4-2 and RE-4-2-AraR.^A731V^ in complex carbon medium. **A**–**F** FPase, *p*NPCase, CMCase, pNPAase, xylanase and pNPXase activities analysis. Data represent mean ± SD from triplicate cultivations. Statistical significance of the differences between strains was calculated for transcriptional levels of endogenous genes. *p < 0.05, **p < 0.01, ***p < 0.001

Strain RE-4-2 and RE-4-2-AraR^A731V^ were cultivated in a complex carbon source medium, and enzyme activity was measured by sampling. As shown in Fig. 5, compared with the starting strain RE-4-2, the filter paper enzyme activity and endonuclease enzyme activity of RE-4-2-AraR^A731V^ were slightly increased, while the exonuclease enzyme activity was significantly increased. In terms of hemicellulase activity, the activities of arabinofuranosidase, xylosidase and xylanase of strain RE-4-2-AraR^A731V^ increased by 7.2 times, 1.2 times and 0.17 times, respectively, compared with the original strain. These results indicate that AraRA731V overexpression can significantly enhance the arabinofuranosidase activity of the strain, and can significantly enhance the xylosidase activity of the strain.

### Saccharification effect of cellulases produced by DB2, RE-3-2, RE-4-2 and RE-5-2 and RE-4-2-AraR^A731V^ on corn stover and corn fiber pretreated with ammonium sulfite

**Fig. 6:**
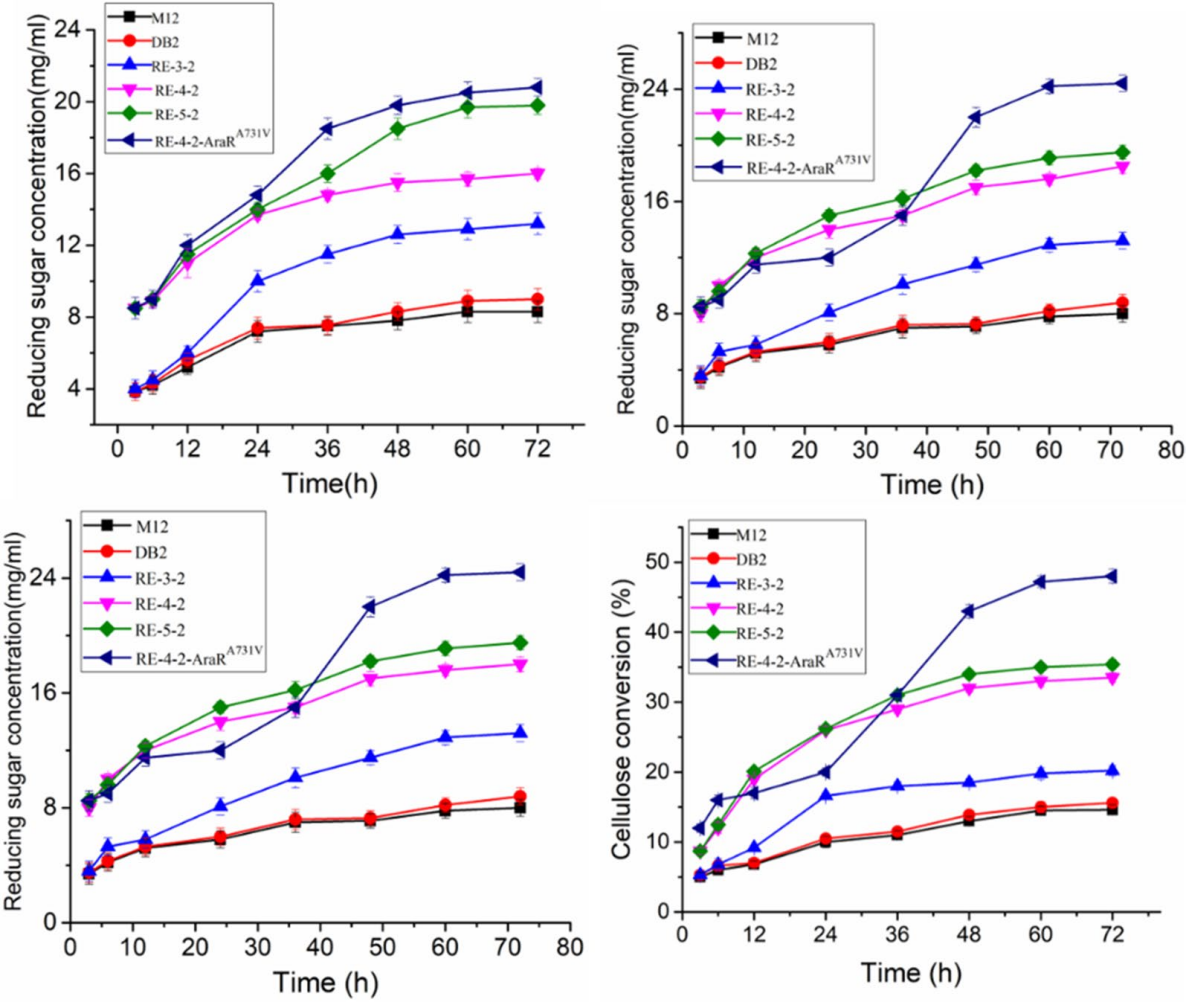
Saccharification effect of cellulases produced by recombinant strains RE-4-2 RE-5-2 and RE-4-2-AraR^A731V^ on corn stover and corn fiber. **A**, **B** Reducing sugar content and cellulose conversion of corn stover; **C**, **D** reducing sugar content and cellulose conversion of corn fiber. Data represent mean ± SD from triplicate cultivations. Statistical significance of the differences between strains was calculated for transcriptional levels of endogenous genes. *p < 0.05, **p < 0.01, ***p < 0.001

The above results indicated that production of lignocellulosic degrading enzymes by recombinant strains were significantly increased. What we are more concern about is that enzymatic hydrolysis efficiency of lignocellulosic biomass by these recombinants were also significantly improved. Corn stover and corn fiber pretreated with ammonium sulfite were used as the substrates and strain RE-4-2 and RE-5-2 with high filter paper enzyme activity and xylanase activity were applied to saccharification experiments. The compositions of corn stover and corn fiber were analyzed after pretreatment with ammonium sulfite. The residual lignin content in the pretreated corn stover and corn fiber was 4.5% and 1.0%, respectively. The content of cellulose in corn fiber and corn stover is 13.3% and 30.2%, and the content of hemicellulose is 45.7% and 34.9% (Additional file 1: Table S2), respectively, which are similar as the previous reports [18–21] and more arabinose in corn fiber than in corn stover (15% vs 3.3%). The fermentation broth of strain M12, RE-4-2, Re-5-2 after six days’ cultivation was prepared for saccharification experiment. The samples were taken at certain time intervals to determine the reducing sugar in the saccharification solution of pretreated corn stover. As shown in Fig. 6, the enzymatic hydrolysis efficiency of strain RE-4-2 on corn stover was nearly twice that of the starting strain M12. After 72 h of enzymatic hydrolysis, the concentration of reducing sugar produced by strain RE-4-2 reached 16.6 mg/mL, which was 93% higher than the 8.6 mg/mL of the original strain M12. The cellulose conversion rate by strain RE-4-2 increased to 28.2%, an increase of 2.82 times compared to M12. RE-5-2 increased cellulose conversion rate to 35.5%, which was 255% higher than M12 and showed an increase of 25.9% on the basis of that of RE-4-2. For the enzymatic hydrolysis of pretreated corn fiber, after 72 h of enzymatic hydrolysis, the concentration of reducing sugar produced by strain RE-4-2 reached 18.5 mg/mL, which was 131% higher than that of the original strain M12. The cellulose conversion rate by strain RE-4-2 also increased from 15.0% of the starting strain M12 to 35.0%, an increase of 1.33 times. However, strain RE-5-2 with extra XLR-1^A828V^ regulatory module of *N. crassa* only showed an increase of approximately 5.0% in reducing sugar yield and cellulose conversion rate compared to strain RE-4-2. The enzymatic hydrolysis efficiency of strain RE-4-2 and RE-5-2 cellulases with equal protein loading was significantly increased. The reason is likely due to the enhancement of transcript levels of cellulolytic enzymes caused by heterologous transcript regulatory module and the increase in the proportion of lignocellulosic degrading enzymes in the total extracellular protein as well as the presence of heterologous cellulase TCBHI in the enzymes (previous studies have shown that the hydrolysis efficiency of cellulose by *T. reesei* CBHI is higher than that of *P. oxalicum* CBHI [22–26]. In conclusion, the modified recombinant strains not only have higher yields of lignocellulosic degrading enzymes, but also have higher enzymatic hydrolysis efficiency for biomass materials such as corn stover and corn fiber.

The saccharification effects of cellulases produced by strain RE-4-2-AraR^A731V^ on pretreated corn stover and corn fiber were further evaluated. After cultivation of 6 days, the cellulases of strain RE-4-2, RE-5-2 and RE-4-2-AraR^A731V^ were applied to saccharification experiments and the results are shown in Fig. 6. For the enzymatic hydrolysis of pretreated corn stover, after 72 h of enzymatic hydrolysis, the reducing sugar yield of strain RE-4-2 was 93% higher than that of M12, and strain RE-5-2 was further increased by 21.1% on the basis of RE-4-2, cellulose conversion reached 35.5%; Strain RE-4-2-AraR^A731V^ carrying point mutated AraR^A731V^ showed an increase in the reducing sugar yield by 33.9% compared to that of strain RE-4-2 and cellulose conversion increased to 38.5%. For the enzymatic hydrolysis of pretreated corn fiber, after 72 h of enzymatic hydrolysis, the reducing sugar yield and cellulose conversion of strain RE-5-2 had a slight increase than those of strain RE-4-2. Strain RE-4-2-AraR^A731V^ carrying point mutated AraR^A731V^ showed a significant improvement in the reducing sugar yield and cellulose conversion compared to strain RE-4-2, with increases of 31.9% and 37%, respectively. This may be related to the fact that the strain RE-4-2-AraR^A731V^ contains more arabinofuranosidase and exocellulase. The content of arabinose in corn stover is about 2.9%, while in corn fiber, the content of arabinose in corn fiber much higher. The cellulases of RE-4-2-AraR^A731V^ are used for enzymatic hydrolysis of corn fiber; hydrolysis efficiency was greatly enhanced.

## Conclusions

Heterologous XlnR homologues have been found to have activity in *P. oxalicum* and have regulatory effects on major cellulases [27]. We also obtained several high lignocellulosic degrading enzyme-producing strain DB2 by knocking out the P*bgl2* gene in the starting strain M12 and strains RE-3-2, RE-4-2, RE-5-2 by accumulative expression of XlnR regulatory modules. Strain RE-3-2 with one regulatory module, RE-4-2 with two regulatory modules and RE-5-2 with three regulatory modules showed increased saccharification efficiency for corn straw and corn fiber and cellulose conversion, which indicated that combining manipulations of regulatory modules including XlnR/Xyr1-Xlr-1 and their target genes is an effective way to increase the degradation lignocellulosic biomass. Strain RE-4-2-AraR^A731V^ constructed by overexpressing AraR^A731V^ in RE-4-2 showed an increase of 7.2 times and 1.2 times in arabinofuranosidase and xylosidase activities, respectively. Reducing sugar yield and cellulose conversion of corn stover and corn fiber by RE-4-2-AraR^A731V^ were further increased, which indicated that the enhancement of enzyme activity of degrading side chain of hemicellulose will be helpful for cellulose degradation.

The specific mechanism by which these regulatory modules exert regulatory effects can be further investigated. This not only will help to understand the mechanism of action of XlnR homologues in heterologous host strains, but also will offer important reference for the heterologous expression of other similar regulatory factors. In addition, other transcript factors, such as cellulase transcriptional activator ClrB and transcriptional inhibitor CreA can be included in the genetic manipulations so that the recombinant strains have higher cellulase production capacity.

### Supplementary Information

**Additional file 1: Table S1.** The primers used in the construction and verification of strains. **Table S2.** Compositions of corn stover and corn fiber (% w/w; based on dry matter). **Figure S1.** PCR verification of recombinant strains RE-3-2, RE-4-2 and RE-5-2. **Figure S2.** The results of partial sequence alignment of XlnR and AraR in *P*. *oxalicum*.

## Data Availability

The datasets generated during and/or analyzed during the current study are not publicly available, but are available from the corresponding author on reasonable request.
