# Supplementary material for: Cumulative expression of heterologous XlnR regulatory modules and AraRA731V in Penicillium oxalicum enhances saccharification efficiency of corn stover and corn fiber
Source: Biotechnol Biofuels Bioprod. 2024 Feb 1;17:18. doi: 10.1186/s13068-024-02464-x (PMC10835966; doi:10.1186/s13068-024-02464-x)
Supplement: Supplementary file 1 — Additional file 1: Table S1. The primers used in the construction and verification of strains. Table S2. Compositions of corn stover and corn fiber (% w/w; based on dry matter). Figure S1. PCR verification of recombinant strains RE-3-2, RE-4-2 and RE-5-2. Figure S2. The results of partial sequence alignment of XlnR and AraR in P. oxalicum. [file 13068_2024_2464_MOESM1_ESM.docx]

**Table S1 The primers used in the construction and verification of strains**

| **DB2-pyrG/DB2**  **construction** | |
| --- | --- |
| bgl2U-F | GGAGCATATTTGTATGGGAG |
| bgl2U-R | CGTAGCCGATCTTGGCCATCTTGGCGAAGTCGATTGGAAC |
| Rec-F | ATGGCCAAGATCGGCTACG |
| Rec-R | GCTAGCGACTGTATCCTTCCAACCAAGAATGGATCGTGGG |
| six-pyrG-F | GGAAGGATACAGTCGCTAGC |
| six-pyrG-R | CGTTCACACGTGAAGC |
| bgl2D-F | GCTTCACGTGTGAACGTGTTCGCTTGATTGCTTG |
| bgl2D-R | AGGAGGTGATCCCAAACA |
| bgl2N-F | GTTCACTGTCCACCATGTCATG |
| bgl2N-R | GTCTCCATGTCTCCATGCAGG |
| **RE-3-1 construction** | |
| PDE_02864-F | CTGGGTTTCTTGCCTGAGTT |
| six-pyrG-R | CGTTCACACGTGAAGC |
| **RE-4-1 construction** | |
| bgl2-F | CAGGGTTCACTGTCCACCATGTCA |
| six-pyrG-R | CGTTCACACGTGAAGC |
| **RE-5-1 construction** | |
| gpdA-F | AGTCAGACGGCGTAACCAAA |
| six-pyrG-R | CGTTCACACGTGAAGC |
| **RE-4-2-AraR^A731V^ construction** | |
| gpdA-F | AGTCAGACGGCGTAACCAAA |
| Hph-R | CAACCCAGGGCTGGTGACGG |
| **RT-qPCR primers** | |
| Actin-SC-F | GTATCATGATTGGTATGGGT |
| Actin-RT-F: | GTTCCATTCTCGCCTCCCTCT |
| Actin-RT-R: | AGAAGCACTTGCGGTGAACGA |
| cbh1-SC-F | CGGCAAGAACTGCTACACTG |
| cbh1-RT-F | CCACCACCACTACCAGCAAGG |
| cbh1-RT-R | GTAGCCAACACCACCGCACT |
| cbh2-SC-F | TGGTGGTATCGGCTACTCTG |
| cbh2-RT-F | CCACCGACACTGGTGATGCT |
| cbh2-RT-R | GCTTGAGGGCATCGCTGTAT |
| eg1-SC-F | CCCGTCCCTGAAGACATACA |
| eg1-RT-F | CCACCACCAAGATTTCCACC |
| eg1-RT-R | GGACACGCAGGCTGTAGGTC |
| Xyn10A-RT-F | GGTCTCCAGGCTCACTTCATC |
| Xyn10A-RT-R | GTCGAGGGCAAGTTCATACG |
| Xyn10A-SC-R | AGGTAGTAGGGCCAGTCCAG |
| Nc-cbh1-SC-F | TTCAGTTCGTCACCAAGGGCTCGT |
| Nc-cbh1-SC-R | AGTTCTCAATGACTTTTCCGTTCTG |
| Nc-cbh1-RT-F | ACGATGCTAACGCTGGTATTGGTGA |
| Nc-cbh1-RT-R | CACAACCATCGGCATCGCAAGTA |
| Nc-eg1-SC-F | AGTGCCCTACTCCAGCATTCATCAA |
| Nc-eg1-SC-R | CGACCCAATCTCCCCACTCTTCACA |
| Nc-eg1-RT-F | CACTCAGTTCTTCAGCAATACGACG |
| Nc-eg1-RT-R | AAGGCGTTGACTCCCCCAAAGAT |
| Tr-cbh1-SC-F | TCACGCTACGAACAGCAGCACGA |
| Tr-cbh1-RT-F | CAACTCCATCTCCGAGGCTCTTACC |
| Tr-cbh1-RT-R | TGGACATAGTATCGGTTGATGGCAC |
| Tr-eg1-SC-F | GACGAGGCGACCTGTGGCAAGA |
| Tr-eg1-SC-R | CTGGCTGTTGTCGTTCCAAATGCT |
| Tr-eg1-RT-F | TCCTCGGCTGTATCTCCTGGACTCT |
| Tr-eg1-RT-R | CGCAGTAGCCGCTCCCGTAGTT |
| bgl2-SC-F | CTCGGTGCTCGGATACAACA |
| bgl2-RT-F | GGCTGATGCGTACACGTTTGA |
| bgl2-RT-R | CGACATAAGTCACGCCGAAGC |
| Po-XlnR-SC-F | GTTGCTGGACAAGGAGTGCG |
| Po-XlnR-RT-F | CGATCCGCTCTTGCCCAGGTA |
| Po-XlnR-RT-R | GGGCGAGAACTTCACGTCTG |
| An-xlnR-SC-F | TCGCTACCCCCGTCCGTCTTAC |
| An-xlnR-SC-R | TTCACTCGTTTGTGCGGCTACCCA |
| An-xlnR-RT-F | ACCGCACCGTTTCTTGGTCTCTC |
| An-xlnR-RT-R | GCTAGCGACTGCGGAATAATGGA |
| Nc-xlr-1-SC-F | CGTGGAAAGGCTTCAAGAAAGGATT |
| Nc-xlr-1-SC-R | CAAATCCAAGGACATAAGGCGACAT |
| Nc-xlr-1-RT-F | CAGTTATGGGAATGTCCACCAAGAA |
| Nc-xlr-1-RT-R | AACCACTTGATGTGTTTGCGGAATA |
| Tr-xyr1-SC-F | TATGTCCGAGAGAGAAAGAAGCGTG |
| Tr-xyr1-SC-R | TGTGCTGATGAAGACGAGGAGAAGT |
| Tr-xyr1-RT-F | ATCAACATCGCAAACTCTCACGC |
| Tr-xyr1-RT-R | GATGACGCTTCCAGCTGAGTTCG |
| Rec-SC-F | CCGCAACAACAAGGAACTGACC |
| Rec-SC-R | CGATTCCACATACAGGGTCTAAAAC |
| Rec-RT-F | TCGCCAAGTCCAAGGGCAAGTT |
| Rec-RT-R | TTGTAGCGGGTGCGGTAGCGG |

Table S2 Compostions of corn stover and corn fiber (% w/w; based on dry matter)

| component | Content (% w/w) based on dry matter | |
| --- | --- | --- |
|  | Corn fiber | Corn stover |
| Monomeric sugars | <1 | <1 |
| starch | 16.0 | 0.5 |
| cellulose | 13.3 | 30.2 |
| Hemicellulose(total) | 45.7 | 34.9 |
| Xylose^a^ | 17.5 | 22.5 |
| Arabinose^a^ | 15.0 | 3.3 |
| Mannose^a^ | 2.0 | 1.8 |
| Galactose^a^ | 3.0 | 1.2 |
| Glucuronic acid^a^ | 2.5 | 2.4 |
| Ferulic acid (ester) | 1.0 | 0.8 |
| Coumaric acid (ester) | 0.2 | 0.4 |
| Acetic acid (ester) | 2.5 | 2.5 |
| Protein | 8.5 | 5.1 |
| Lignin | 1.0 | 4.5 |

^a^ Presented as polymers;

Fig. S1


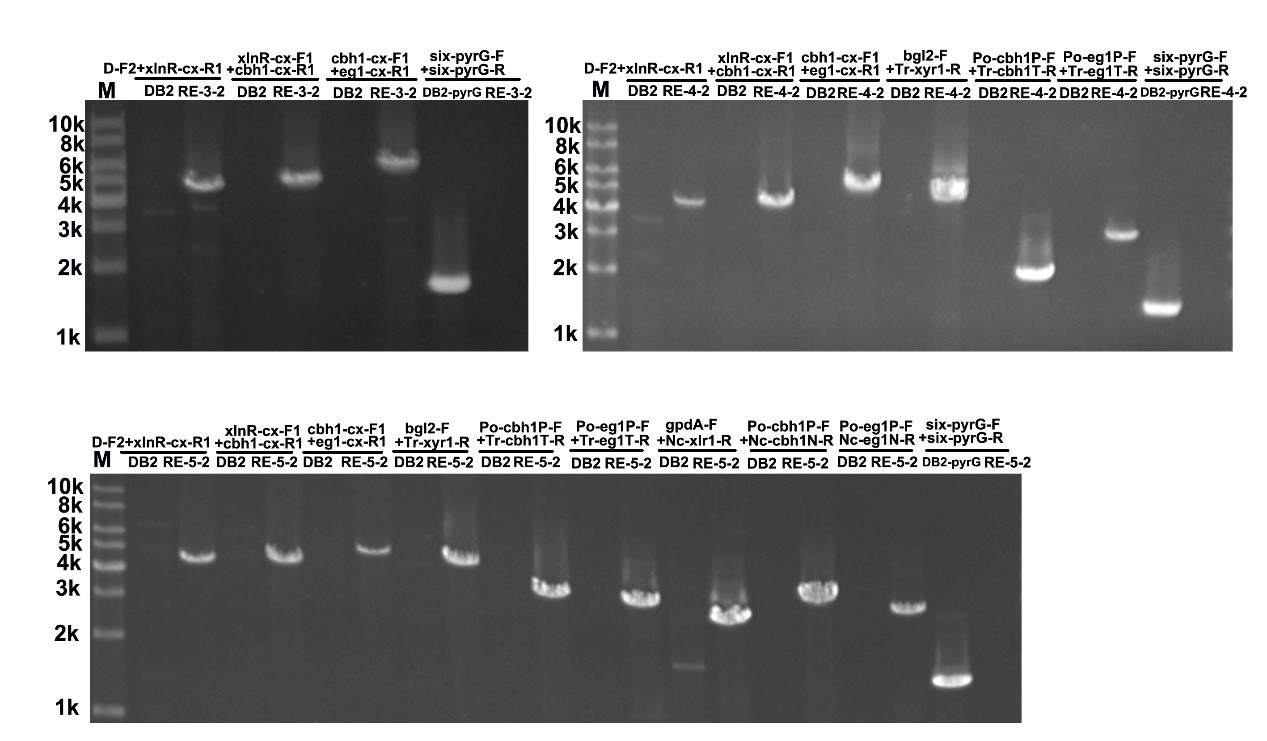


Fig. S1 PCR verification of recombinant strains RE-3-2, RE-4-2 and RE-5-2

Fig. S2


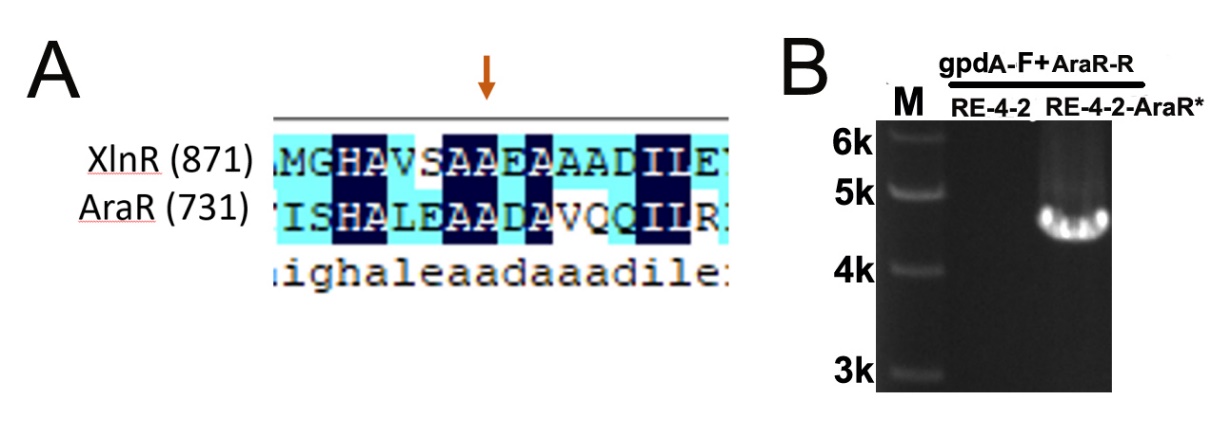


Fig. S2 The results of partial sequence alignment of XlnR and AraR in *P. oxalicum*
